# A novel GAA-repeat-expansion-based mouse model of Friedreich’s ataxia

**DOI:** 10.1242/dmm.018952

**Published:** 2015-02-13

**Authors:** Sara Anjomani Virmouni, Vahid Ezzatizadeh, Chiranjeevi Sandi, Madhavi Sandi, Sahar Al-Mahdawi, Yogesh Chutake, Mark A. Pook

**Affiliations:** 1 Ataxia Research Group, Division of Biosciences, Department of Life Sciences, College of Health & Life Sciences, Brunel University London, Uxbridge, UB8 3PH, UK; 2 Synthetic Biology Theme, Institute of Environment, Health and Societies, Brunel University London, Uxbridge, UB8 3PH, UK; 3 Department of Pediatrics, Section of Genetics, University of Oklahoma Health Sciences Center, Oklahoma City, OK 73104, USA

**Keywords:** GAA repeat, Friedreich’s ataxia, FRDA, YG8sR, Mouse model

## Abstract

Friedreich’s ataxia (FRDA) is an autosomal recessive neurodegenerative disorder caused by a GAA repeat expansion mutation within intron 1 of the *FXN* gene, resulting in reduced levels of frataxin protein. We have previously reported the generation of human *FXN* yeast artificial chromosome (YAC) transgenic FRDA mouse models containing 90–190 GAA repeats, but the presence of multiple GAA repeats within these mice is considered suboptimal. We now describe the cellular, molecular and behavioural characterisation of a newly developed YAC transgenic FRDA mouse model, designated YG8sR, which we have shown by DNA sequencing to contain a single pure GAA repeat expansion. The founder YG8sR mouse contained 120 GAA repeats but, due to intergenerational expansion, we have now established a colony of YG8sR mice that contain ~200 GAA repeats. We show that YG8sR mice have a single copy of the *FXN* transgene, which is integrated at a single site as confirmed by fluorescence *in situ* hybridisation (FISH) analysis of metaphase and interphase chromosomes. We have identified significant behavioural deficits, together with a degree of glucose intolerance and insulin hypersensitivity, in YG8sR FRDA mice compared with control Y47R and wild-type (WT) mice. We have also detected increased somatic GAA repeat instability in the brain and cerebellum of YG8sR mice, together with significantly reduced expression of *FXN*, *FAST-1* and frataxin, and reduced aconitase activity, compared with Y47R mice. Furthermore, we have confirmed the presence of pathological vacuoles within neurons of the dorsal root ganglia (DRG) of YG8sR mice. These novel GAA-repeat-expansion-based YAC transgenic FRDA mice, which exhibit progressive FRDA-like pathology, represent an excellent model for the investigation of FRDA disease mechanisms and therapy.

## INTRODUCTION

Friedreich’s ataxia (FRDA) is an autosomal recessive disorder, characterised by progressive neurodegeneration, cardiomyopathy, diabetes mellitus and skeletal deformities (Pandolfo, 2009). It is primarily caused by a GAA repeat expansion mutation within intron 1 of the *FXN* gene, resulting in reduced levels of frataxin protein (Campuzano et al., 1997; Campuzano et al., 1996). Unaffected individuals have 5 to 40 GAA repeat sequences, whereas affected individuals have ~70 to more than 1000 GAA triplets (Pandolfo, 2002). Frataxin is a mitochondrial protein involved in iron-sulphur cluster and heme biosynthesis (Gerber et al., 2003). Reduction in frataxin expression leads to oxidative stress, mitochondrial iron accumulation and consequential cell death, with the primary sites being large sensory neurons of the dorsal root ganglia (DRG) and the dentate nucleus of the cerebellum (Campuzano et al., 1997; Koeppen, 2011). Although FRDA is the most common inherited ataxia, affecting 1 in 50,000 Caucasians, there is currently no effective treatment. Therefore, to investigate FRDA molecular disease mechanisms and therapy, a number of different FRDA cell and mouse models have been developed (Perdomini et al., 2013). Our lab have previously established three human *FXN* YAC transgenic mouse models that express human *FXN* in a mouse-*Fxn*-null background: Y47R, containing normal-sized (GAA)_9_ repeats, and YG8R and YG22R, which initially contained expanded GAA repeats of 90–190 units and 190 units, respectively, but which have subsequently been bred to contain expanded GAA repeats of 120–220 units and 170–260 units, respectively (Al-Mahdawi et al., 2004; Al-Mahdawi et al., 2006; Anjomani Virmouni et al., 2014; Pook et al., 2001). We have demonstrated that both YG8R and YG22R FRDA mice express comparatively decreased levels of human *FXN* mRNA and frataxin protein in comparison to wild-type (WT) or Y47R control mice (Al-Mahdawi et al., 2008; Al-Mahdawi et al., 2006; Anjomani Virmouni et al., 2014). Furthermore, both YG8R and YG22R mice exhibit a progressive FRDA-like molecular disease phenotype, which includes intergenerational and somatic instability of the GAA repeat expansion mutation (Al-Mahdawi et al., 2004; Clark et al., 2007), as well as mild progressive behavioural motor coordination deficits, compared with WT or Y47R controls, that are consistent with FRDA disease (Al-Mahdawi et al., 2006; Anjomani Virmouni et al., 2014). In this study, we report the generation of a new line of GAA-repeat-expansion-based FRDA mice derived from YG8R breeding, designated YG8sR, which contains a single copy of the *FXN* transgene and a single pure GAA repeat expansion mutation, which was 120 GAA repeats in size in the founder mouse. The GAA repeat remains as a single unit upon transmission, but exhibits both intergenerational and somatic variability in repeat size. We demonstrate progressive behavioural deficits in YG8sR mice, together with significant decreases of *FXN* and *FAST-1* transcripts and frataxin protein expression compared with C57BL6/J (B6) WT and Y47R controls. In addition, the YG8sR mice exhibited pathology of the DRG, revealed by the presence of numerous vacuoles within the large sensory neuronal cell bodies, together with reduced levels of brain aconitase activity, in line with an FRDA-like phenotype. Therefore, these YG8sR mice currently represent the most suitable GAA-repeat-based YAC transgenic mouse model to investigate potential FRDA therapies. These mice are available from The Jackson Laboratory: YG8sR (#024113).

**TRANSLATIONAL IMPACT**

**Clinical issue**

Friedreich’s ataxia (FRDA) is an inherited neurodegenerative disorder that also affects the heart and pancreas. It is the most common hereditary ataxia, affecting approximately 1 in 50,000 individuals in the Caucasian population. It is caused by a GAA repeat expansion mutation within intron 1 of the *FXN* gene, which results in decreased expression of frataxin, the essential mitochondrial protein that this gene encodes. At present, there is no therapy for FRDA; therefore, much research effort is currently focused on the development of optimal cell and animal models of FRDA for preclinical therapeutic testing. The YG8R mouse model of FRDA – *FXN*-knockout mice that are also hemizygous for the YG8 transgene (which carries two tandem copies of the human *FXN* gene with two GAA trinucleotide repeat expansions) – has been used to successfully test the safety and efficacy of frataxin-increasing drug compounds, such as histone deacetylase inhibitors and interferon-γ. However, it is not an optimal model because it contains multiple GAA repeat stretches, does not express particularly low levels of frataxin and has a very mild overall phenotype, which is not conducive to effective preclinical testing.

**Results**

Here, the authors report on the development and characterisation of a novel FRDA mouse model from YG8R breeding, designated YG8sR. PCR genotyping analysis and DNA sequencing showed that YG8sR mice contain a single GAA repeat expansion mutation, and that this expansion has both intergenerational and somatic instability, as is detected in humans with FRDA. These mice also have significantly decreased levels of frataxin expression in all tissues tested and show FRDA-like phenotypes such as defects in coordination behaviour, reduced brain aconitase enzyme activity and pathological dorsal root ganglia histology.

**Implications and future directions**

These novel YG8sR GAA-repeat-expansion-based human *FXN* transgenic mice represent an excellent model for the investigation of FRDA disease mechanisms and therapy, adding to and optimising the currently available models for this neurodegenerative disease. YG8sR mice will be particularly useful for any FRDA therapeutic strategies using compounds that target the mutated human *FXN* gene sequence to increase frataxin expression, such as DNA or RNA oligotherapies. Additionally, to assist future FRDA therapeutic preclinical studies, the authors aim to continue selectively breeding YG8sR mice to produce ever larger GAA repeat expansions that cause further reductions in frataxin expression. Other laboratories are also making efforts to generate similar human *FXN* BAC transgenic mice with very large pure GAA repeats. Future studies using the YG8sR, and subsequent, models could be highly important given the current lack of a therapy for this devastating disease.

## RESULTS

### Identification of YG8sR mice with an *FXN* transgene containing a single pure GAA repeat sequence

**Fig. 1.**
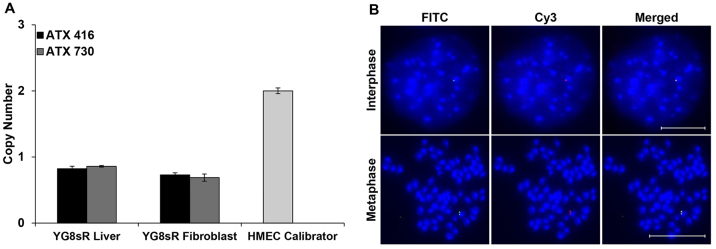
**Transgene copy number.** (A) Two TaqMan copy-number assays were applied: Hs05092416-cn assay (ATX 416; represented in black) was designed to amplify a 106-bp fragment of *FXN* within intron 3, and Hs02407730-cn assay (ATX 730; represented in grey) was designed to amplify an 80-bp fragment of *FXN* within intron 1 and exon 2. Human mammary epithelial cell (HMEC) with copy numbers of two served as a calibrator. Error bars=s.d. *n*=2. (B) Determination of the integration site of the transgenic *FXN* gene by FISH. Biotin-labelled RP11-265B8 and digoxigenin-labelled RP11-876N18 probes were hybridised onto interphase and metaphase chromosomes (DAPI stained) of YG8sR cells. YG8sR showed one hybridisation signal, indicating a single integration site of the *FXN* transgene containing one copy of the *FXN* gene. Scale bars: 10 μm.

During normal breeding of previously characterised YG8R *FXN* YAC transgenic mice, which contain multiple GAA repeats (Al-Mahdawi et al., 2004; Al-Mahdawi et al., 2006), we serendipitously identified a unique founder mouse that contained a single GAA repeat sequence of 120 units (supplementary material Fig. S1). The YAC transgene has previously been shown by gene scanning at The Jackson Laboratory to be integrated into mouse chromosome 16 (C. Lutz, The Jackson Laboratory, personal communication). From this founder mouse, we established a new line of GAA-repeat-expansion-containing *FXN* YAC transgenic mice on a predominantly C57BL6/J background, designated YG8sR. We observed that the GAA repeat was transmitted from parent to offspring as a single discrete entity, but with variation of the specific GAA repeat size. Therefore, by selecting for intergenerational GAA repeat expansion events, we have been able to establish a colony of YG8sR mice that now contain GAA repeats of ~200 units (supplementary material Fig. S1). The single GAA repeat was shown to be a pure GAA repeat, firstly by *Mbo*II digestion (Holloway et al., 2011) and then by DNA sequencing analysis (supplementary material Fig. S2). The *FXN* transgene copy number was then investigated in YG8sR mouse genomic DNA samples using TaqMan real-time PCR. The results indicated that YG8sR mice had a single copy of the *FXN* transgene (Fig. 1A). In addition, to determine the integration site of the transgenic *FXN* gene and to confirm the TaqMan copy-number results, fluorescence *in situ* hybridisation (FISH) using dual-colour probes containing overlapping bacterial artificial chromosome (BAC; RP11-265B8 and RP11-876N18) sequences was performed on both metaphase and interphase spreads of YG8sR cultured fibroblast cells. The YG8sR cells showed one hybridisation signal, indicating the presence of a single integration site containing one copy of the *FXN* transgene (Fig. 1B).

### YG8sR mice exhibit behavioural deficits

**Fig. 2.**
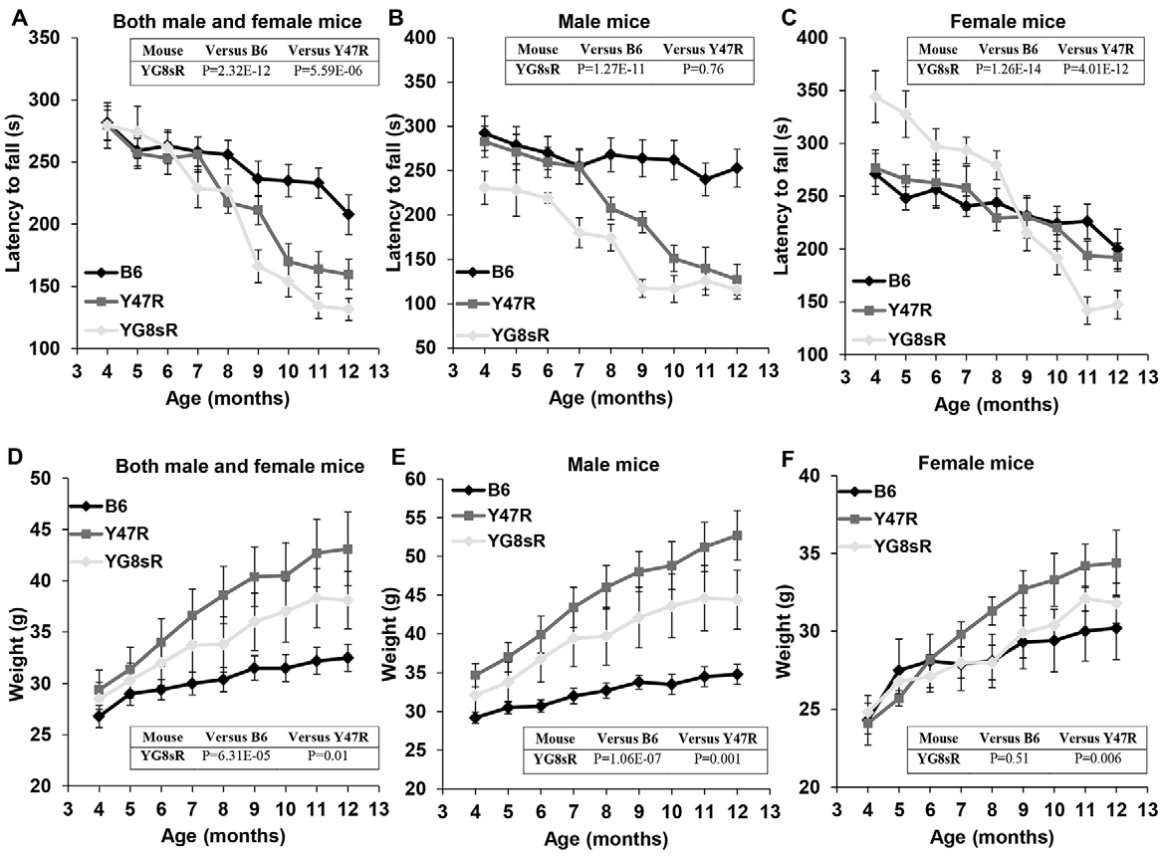
**Rotarod and weight analysis.** YG8sR FRDA mice show a coordination deficit compared with B6 and Y47R controls when (A) both male and female values are taken together (*n*=10 mice per genotype), or when results are for (B) males alone (*n*=5 mice per genotype) or (C) females alone (*n*=5 mice per genotype). (D) Weight analysis of YG8sR compared with B6 and Y47R controls when both male and female values were taken together (*n*=10 mice per genotype). The results indicated a significant increase in weight of YG8sR FRDA mice in comparison to B6 control. A similar tendency was seen when (E) male and (F) female values were analysed separately (*n*=5 mice per genotype). Values represent mean±s.e.m.

The motor coordination ability of YG8sR FRDA mice was assessed on a rotarod treadmill at monthly time points from 4–12 months of age, using B6 and Y47R mice as controls. Ten mice were assessed for each group, five males and five females. As shown in Fig. 2, the coordination ability of the YG8sR mice was significantly reduced when compared with B6 and Y47R controls. This trend held true when both male and female values were taken together (Fig. 2A) or when male and female values were considered alone (Fig. 2B,C). However, no significant difference was detected in the rotarod performance of YG8sR males compared with Y47R controls. This might be due to the higher body weight of Y47R control mice, affecting their rotarod performance (Fig. 2D–F). YG8sR mice were lighter than Y47R controls, but heavier than B6 controls, when both male and female values were taken together (Fig. 2D) or when male values were considered alone (Fig. 2E), but there was no statistically significant difference between YG8sR and B6 females (Fig. 2F).

**Fig. 3.**
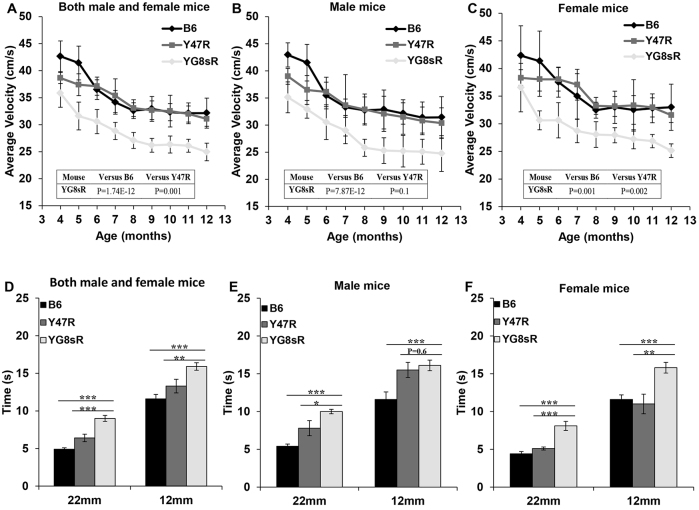
**Average velocity and beam-walk analysis of YG8sR FRDA mice.** (A–C) Average velocity. (A) YG8sR displayed significantly decreased average velocity compared with B6 and Y47R controls when both male and female values were taken together (*n*=10 mice per genotype). Analysis of (B) males and (C) females separately (*n*=5 mice per genotype). (D–F) Beam walk. (D) Analysis of YG8sR compared to B6 and Y47R controls showed a coordination deficit on both 22-mm and 12-mm beams (*n*=10 mice per genotype) when both male and female values were taken together. Analysis of (E) males and (F) females separately (*n*=5 mice per genotype). Values represent mean±s.e.m. *P<0.05, **P<0.01 and ***P<0.001. Statistical differences between YG8sR FRDA and B6 control mice are indicated by the top bar, whereas the bottom bar indicates the differences between YG8sR FRDA and Y47R control mice.

Locomotor activity tests were performed over a 2-minute period and repeated four times for each mouse using an open-field beam-breaker activity monitor. Ten mice (five males and five females) were assessed monthly for each group from 4–12 months of age. YG8sR mice exhibited significantly reduced average velocity (total distance covered divided by the total time elapsed) compared with B6 and Y47R controls, whether male and female values were taken together or analysed separately (Fig. 3A–C). The ambulatory distance (total distance covered by the mice within a specific time) of YG8sR FRDA mice was significantly less than the controls when male and female values were taken together or when males were analysed separately, but higher ambulatory values were observed in YG8sR females (supplementary material Fig. S3 and Table S1). Significant decreases in the vertical count and vertical time (total events and duration of the mouse standing on hind legs) were detected in the YG8sR mice compared with the controls when analysing males and females together and separately (supplementary material Fig. S4 and Table S1). Significant decreases in jump count and jump time were also detected in the YG8sR mice compared with the controls when analysing males and females together or males separately, but the female-only values showed no statistically significant difference to controls (supplementary material Fig. S5 and Table S1).

Beam-walk performance testing was used to assess the coordination ability of 12-month-old YG8sR FRDA mice compared with B6 and Y47R controls. Ten mice (five males and five females) were assessed for each group and the average latency of four trials on beams of 22-mm and 12-mm diameter was recorded. As evident in Fig. 3D–F, YG8sR mice took significantly longer to cross either the 22-mm or the 12-mm beam compared with the controls when analysing males and females together and separately, although no significant difference was detected in the beam-walk performance of YG8sR males on the 12-mm beam compared with Y47R controls. This might be due to the higher body weight of Y47R male mice (Fig. 2E), affecting their balance and performance on the narrower beam.

**Fig. 4.**
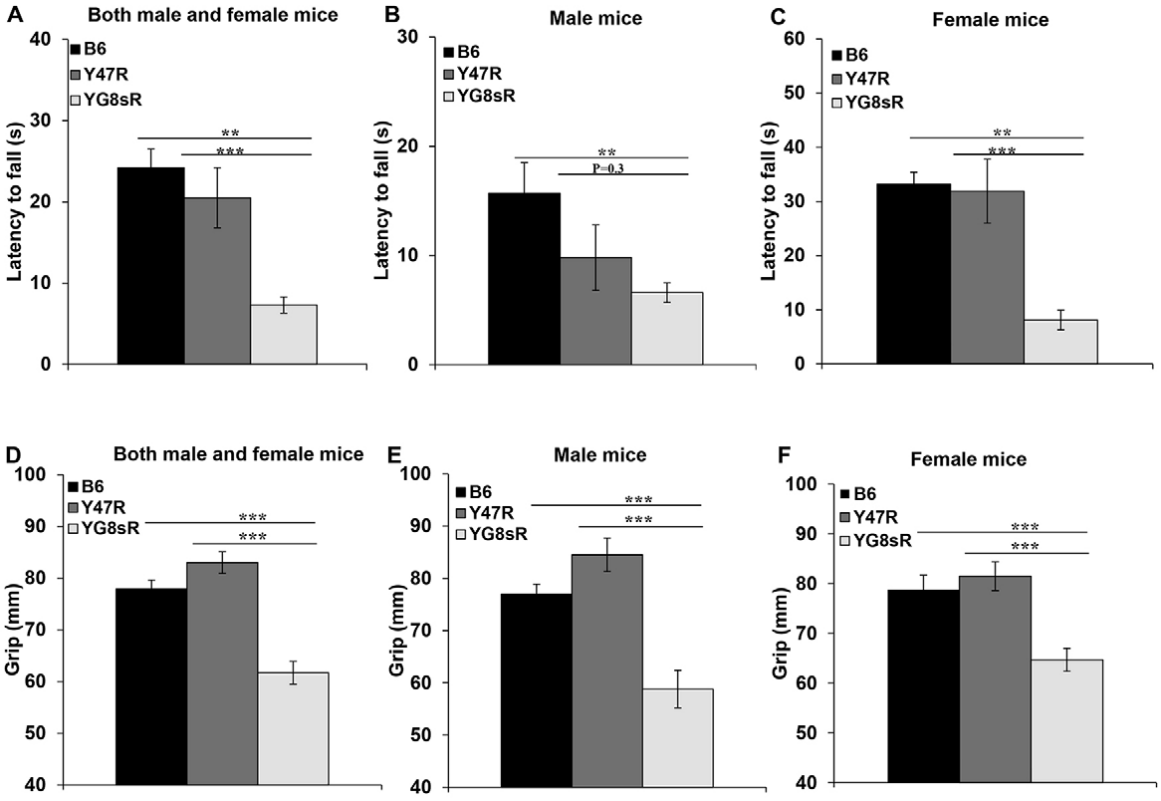
**Hang-wire and grip strength analysis.** (A–C) Hang wire. (A) Analysis of YG8sR revealed impaired neuromuscular strength and lack of coordinated motor control compared with B6 and Y47R controls when both male and female values were taken together (*n*=10 mice per genotype). Analysis of (B) males and (C) females separately (*n*=5 mice per genotype). (D–F) Grip strength. (D) Analysis of YG8sR mice revealed significantly reduced grip strength compared with B6 and Y47R controls when both males and females were analysed together (*n*=10 mice per genotype). Analysis of (E) males and (F) females separately (*n*=5 mice per genotype). Values represent mean±s.e.m. **P<0.01 and ***P<0.001. Statistical differences between YG8sR FRDA and B6 control mice are indicated by the top bar, whereas the bottom bar indicates the differences between YG8sR FRDA and Y47R control mice.

Groups of ten (five male and five female) 12-month-old mice were assessed for their ability to hang on to a horizontal wire by their forepaws. YG8sR mice fell off the wire quicker when compared with Y47R and B6 controls, suggesting a reduced overall forelimb strength (Fig. 4A–C). Statistically significant differences were obtained for all comparisons, with the exception of YG8sR males versus Y47R control male mice, which might be due to the Y47R male mice being heavier than YG8sR males (Fig. 2E), so having a reduced ability to hang on to the wire for any extended period of time. To further assess forelimb grip strength, a grip strength meter was used to measure the peak force with which mice pulled a wire. Ten 12-month-old mice were assessed for each group (five males and five females). The results showed statistically significant decreases in grip strength of YG8sR mice compared with both Y47R and B6 controls, whether both male and female values were taken together or when male and female values were considered alone (Fig. 4D–F).

Gait abnormalities were assessed by monitoring the footprint patterns of 12-month-old mice after they had walked along a narrow gangway. Analysis of the footprints indicated statistically significant decreases in both stride length and base width of YG8sR mice compared with Y47R and B6 controls, whether both male and female values were taken together or when male and female values were considered alone (supplementary material Fig. S6).

### YG8sR mice exhibit glucose intolerance

**Fig. 5.**
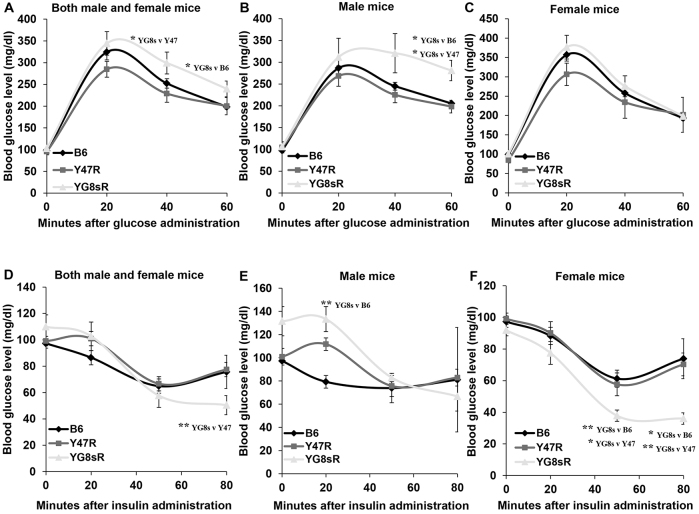
**Glucose- and insulin-tolerance tests.** (A–C) Glucose-tolerance test. (A) Glucose concentration was higher in YG8sR compared with B6 and Y47R controls when both male and female values were taken together (*n*=10 mice per genotype). (B) Similar results were obtained when male values were considered alone (*n*=5 mice per genotype). (C) Analysis of female mice showed no statistically significant difference between the YG8sR FRDA and control mice (*n*=5 mice per genotype). (D–F) Insulin-tolerance test. (D) YG8sR showed lower blood glucose level after insulin injection compared with B6 and Y47R controls when both male and female values were considered. (E) Although the blood glucose concentration was normalised after 50 minutes, YG8sR FRDA male mice exhibited a more rapid glucose lowering after insulin injection. (F) Female mice showed a greater reduction in blood glucose concentration after 50 minutes. Values represent mean±s.e.m. *P<0.05 and **P<0.01.

A fasting glucose-tolerance test was performed on groups of ten (five male and five female) 12-month-old mice, revealing higher blood glucose concentrations in YG8sR mice compared with Y47R and B6 controls, indicating a degree of glucose intolerance in YG8sR mice (Fig. 5A–C). This effect was more pronounced in males compared to females. Subsequent insulin-tolerance testing showed that YG8sR mice had enhanced glucose utilisation after insulin injection compared with controls, suggesting insulin hypersensitivity (Fig. 5D–F).

### YG8sR mice exhibit somatic GAA-repeat instability together with reduced *FXN*, frataxin and *FAST-1* expression in YG8sR tissues

**Fig. 6.**
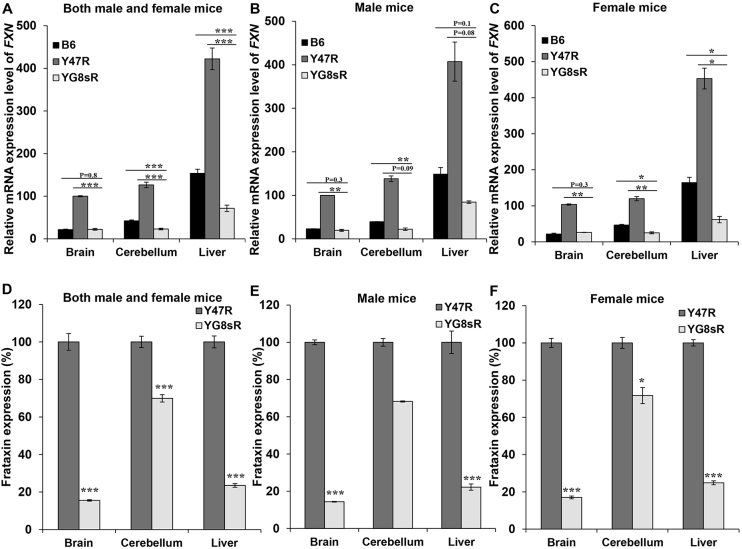
**Frataxin expression levels.** (A–C) qRT-PCR analysis of transgenic *FXN* mRNA using mouse-human specific primers. (A) Analysis of males and females together. Analysis of (B) males and (C) females separately. Data were normalised to the mean *FXN* mRNA level of Y47R brain samples taken as 100%. Statistical differences between the YG8sR mutant and B6 controls are indicated by the top bar, whereas the bottom bar indicates the differences between the YG8sR mutant and Y47R controls. (D–F) Dipstick immunoassay of human frataxin protein. (D) Analysis of males and females together, or (E) males and (F) females separately. Data were normalised to the mean frataxin level of Y47R samples taken as 100%. Values represent mean±s.e.m. *P<0.05, **P<0.01 and ***P<0.001. *n*=4–8.

**Fig. 7.**
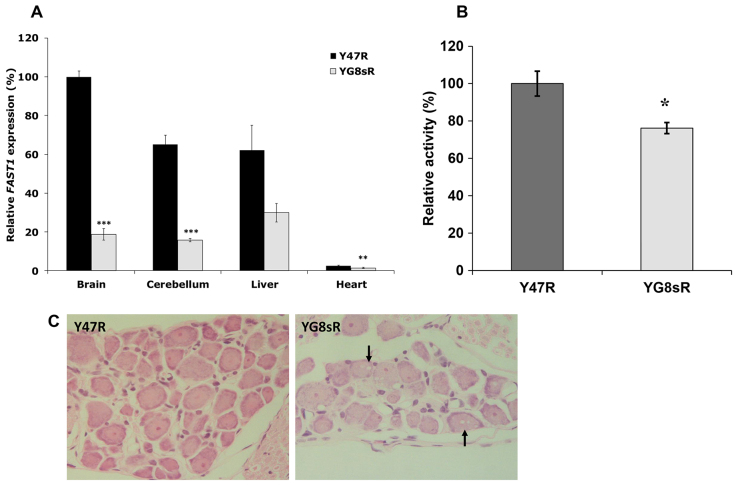
***FAST-1* expression, aconitase activity and DRG pathology.** (A) qRT-PCR analysis of *FAST-1* expression. Data were normalised to the mean *FAST-1* level of Y47R brain samples taken as 100%. Values represent mean±s.e.m. **P<0.01, ***P<0.001. (B) Aconitase activity assays were performed twice on two samples in triplicate with values being calculated relative to citrate synthase activities. Activity ratios are shown relative to the Y47R mouse cell value taken as 100%. Values represent mean±s.e.m. *P<0.05. (C) H&E staining of DRG sections from Y47R and YG8sR FRDA mice. Arrows show vacuolar degeneration in the cytoplasm of DRG neurons from YG8sR mice.

Somatic GAA-repeat instability was assessed by PCR analysis of genomic DNA from a variety of tissues from 12-month-old YG8sR mice. Our analysis revealed tissue-selective GAA repeat expansions, ranging from ~120 to 150 repeats, in the brain, cerebellum and liver tissues, but most prominently in the brain and cerebellum (supplementary material Fig. S7). To assess the effect of the GAA repeat expansion on *FXN* mRNA expression in the YG8sR FRDA mice, qRT-PCR measurements were performed using primers designed to detect both human and mouse *FXN* cDNA. Analysis of the YG8sR mice (both male and female) revealed a very significant reduction of *FXN* mRNA in the brain (22%, *P*<0.001), cerebellum (18%, *P*<0.001) and liver (17%, *P*<0.001) tissues compared with Y47R control mice, which have previously been shown to contain one copy of the human transgene and relatively high *FXN* expression of unknown cause compared with B6 mice (Anjomani Virmouni et al., 2014) (Fig. 6A). In addition, The *FXN* mRNA expression levels were also decreased to 47% (*P*<0.001) in the liver and 55% (*P*<0.001) in the cerebellum of the YG8sR compared with the B6 control (Fig. 6A). On the other hand, analysis of the male YG8sR mice revealed reduced *FXN* mRNA levels of 20% (*P*<0.01) and 85% (*P*=0.3) in the brain, 16% (*P*<0.01) and 56% (*P*=0.09) in the cerebellum, and 21% (*P*=0.08) and 57% (*P*=0.1) in the liver compared with Y47R and B6 controls, respectively (Fig. 6B). Furthermore, the levels of transgenic *FXN* mRNA expression in YG8sR females were decreased to 25% (*P*<0.01) in the brain, 21% (*P*<0.01) in the cerebellum and 14% (*P*<0.05) in the liver tissues (Fig. 6C). The *FXN* expression levels were also decreased in cerebellum (53%, *P*<0.05) and liver (38%, *P*<0.05) of YG8sR females compared with endogenous B6 *Fxn* mRNA (Fig. 6C). To determine the levels of human frataxin expression in the YG8sR FRDA mouse model, frataxin protein expression levels were measured by lateral flow immunoassay with the Frataxin Protein Quantity Dipstick assay kit. Analysis of YG8sR males and females together revealed that frataxin expression was significantly decreased to ~16% (*P*<0.001) in the brain, 70% (*P*<0.001) in the cerebellum and 24% (*P*<0.001) in the liver tissues compared with Y47R controls (Fig. 6D). Males and females were also analysed separately in order to determine the gender-specific differences in the FXN expression level. The results from the males revealed a significant decrease of FXN expression in the brain (14%, *P*<0.001), and also in the liver (22%, *P*<0.001) of YG8sR mice (Fig. 6E). The same trend was also observed in the cerebellum of YG8sR males (69%, *P*=0.08); however, the differences did not reach a statistical significance (Fig. 6E). Analysis of the females showed a marked reduction of FXN expression in the brain (17%, *P*<0.001), cerebellum (72%, *P*<0.05) and liver (25%, *P*<0.001) of YG8sR female mice (Fig. 6F). However, because this approach uses a human-specific anti-frataxin antibody, it did not allow comparison of the human frataxin levels in the YG8sR FRDA transgenic mice with B6 mouse frataxin levels. Levels of the human-specific frataxin antisense transcript, *FAST-1*, were also measured by qRT-PCR and were found to be significantly decreased in YG8sR mouse tissues compared with Y47R controls (Fig. 7A).

### YG8sR mice show decreased aconitase activity and DRG histopathology

Aconitase enzyme activities were measured in the YG8sR and Y47R control brain tissue total cell lysates. Our results showed significantly decreased aconitase activity in the YG8sR brain tissue (76%, *P*<0.05) compared with in Y47R controls (Fig. 7B). Hematoxylin and eosin (H&E) staining of DRG from 12-month-old mice revealed numerous pathological vacuoles within the large neuronal cell bodies of YG8sR mice, present in 17% of all observed large cells, indicative of the FRDA-like disease phenotype, whereas no such vacuoles were present in the Y47R control mice (Fig. 7C).

## DISCUSSION

FRDA is a lethal inherited disorder for which there is no effective therapy. Therefore, there is great interest in developing new tools for FRDA therapeutic testing, including the development of FRDA mouse models. We have previously generated YG8R and YG22R FRDA mouse models (Al-Mahdawi et al., 2006; Anjomani Virmouni et al., 2014) that are based upon human *FXN* YAC transgenic mice containing a large *FXN* genomic transgene with GAA repeat expansions (Al-Mahdawi et al., 2004) crossed with *Fxn*-knockout (KO) mice (Cossée et al., 2000). These FRDA mouse models have proved useful in studies of FRDA disease mechanisms (Chan et al., 2013; Hayashi et al., 2014; Sahdeo et al., 2014; Shan et al., 2013), potential novel FRDA drug therapies (Li et al., 2013; Sandi et al., 2011; Tomassini et al., 2012) and cell-based therapeutic research (Jones et al., 2013; Jones et al., 2015). However, both YG8R and YG22R mice have multiple GAA repeat sequences in the human *FXN* transgene, which complicates their use in studying the effects of GAA repeat expansion disease mechanisms and therapies. Furthermore, both YG8R and YG22R mice have only moderate reductions in frataxin expression and a resultant mild progressive FRDA-like disease phenotype. Therefore, it was considered most beneficial to develop further human-GAA-repeat-expansion-based FRDA mouse models. We now report the development and characterisation of a new FRDA mouse model, designated YG8sR, derived from natural breeding of YG8R mice. YG8sR mice contain a single GAA repeat sequence within a single *FXN* transgene, originally 120 GAA repeat units in size in the founder mouse but, by selective breeding of further GAA-repeat-expansion mice, we have now established a colony of YG8sR mice with 200 GAA repeats.

An important aspect of the YG8sR mice is that they contain a single pure GAA repeat expansion, as we have determined by DNA sequencing, and this GAA repeat exhibits typical somatic GAA repeat instability in brain and cerebellum tissues. This is important because pure GAA repeat expansions are typically identified in humans with FRDA and they might be essential for somatic GAA-repeat-instability-associated disease progression (Montermini et al., 1997), whereas interrupted GAA repeats might induce repeat stability and less severe *FXN* gene silencing effects, resulting in a milder late-onset FRDA phenotype (McDaniel et al., 2001; Stolle et al., 2008). Furthermore, interruptions in other trinucleotide-repeat disease mouse models, such as the BACHD and YAC 128 CAG repeat Huntington disease (HD) transgenic mice, have prevented the development of a somatic repeat instability phenotype in these mice (Pouladi et al., 2012), in contrast to three different HD CAG knock-in (KI) mouse models that all have pure CAG repeats and exhibit extensive CAG repeat instability (Ishiguro et al., 2001; Shelbourne et al., 1999; Wheeler et al., 1999).

Another important aspect of the YG8sR mice is that they express significantly less *FXN* mRNA and frataxin protein in tissues compared with the values previously reported for YG8R and YG22R mice (Al-Mahdawi et al., 2008; Al-Mahdawi et al., 2006; Anjomani Virmouni et al., 2014). However, this translates into only a slightly more severe FRDA-like behavioural, biochemical and histopathological phenotype for YG8sR mice compared with YG8R and YG22R mice; the YG8sR disease phenotype remains slowly progressive. Therefore, to assist future FRDA therapeutic preclinical studies we aim to continue selectively breeding YG8sR mice to produce ever larger GAA repeat expansions leading to further reductions in frataxin expression, while other laboratories are also making efforts to generate similar human *FXN* BAC transgenic mice with very large pure GAA repeats. At the same time as measuring *FXN* expression levels, we also investigated levels of *FAST-1* transcripts in YG8sR tissues compared with those in Y47R controls, and we identified similar significantly decreased levels of both *FXN* and *FAST-1* expression in all tissues. This is different to previous *in vitro* studies carried out by our lab and others of human and mouse fibroblasts; in these studies, FRDA cells exhibited increased levels of *FAST-1* expression relative to control cells (De Biase et al., 2009; Sandi et al., 2014). Therefore, further studies will be required to determine the role, if any, of *FAST-1* expression in relation to *FXN* expression and FRDA. Our finding of pathological vacuoles within the large sensory neuronal cell bodies of the DRG of YG8sR mice is consistent with the initial report by Simon and colleagues, who identified such vacuoles within their prion-promoter-driven conditional frataxin-KO mice (Simon et al., 2004), and also with our own investigations of YG8R and YG22R mice (Al-Mahdawi et al., 2006; Sandi et al., 2011; Tomassini et al., 2012). However, such large vacuoles have not been evident in DRG pathological autopsy specimens from humans with FRDA, which rather exhibit selective neuronal cell loss (Koeppen et al., 2009). Therefore, our findings in FRDA mice could be consistent with an early-stage neuronal cell pathology that perhaps involves autophagy of damaged mitochondria (mitophagy) before progressing to more complete neuronal degeneration.

YG8sR mice represent one of the many different approaches to establish effective FRDA mouse models, which also include conditional frataxin-KO models (Martelli et al., 2012; Puccio et al., 2001; Ristow et al., 2003; Simon et al., 2004; Thierbach et al., 2005) and GAA-repeat KI and KI/KO (KIKO) models (Coppola et al., 2006; Miranda et al., 2002). The conditional frataxin-KO models, which exhibit severe early-onset FRDA-like pathologies, have been extremely useful for assessing potential FRDA drug therapies acting on the downstream effects of frataxin deficiency, including antioxidants and iron chelators (Seznec et al., 2004; Whitnall et al., 2008), and more recently for FRDA gene therapy (Perdomini et al., 2014). In contrast, the KIKI and KIKO models, which exhibit GAA-repeat-induced frataxin deficiency but only a mild FRDA-like phenotype, have been more useful to investigate epigenetic-based frataxin-increasing drug therapies, such as histone deacetylase (HDAC) inhibitors (Rai et al., 2010; Rai et al., 2008). We consider that our YG8sR mice will also continue to be useful for such epigenetic-based frataxin-increasing drug studies, as YG8R mice have previously been for HDAC inhibitor studies (Sandi et al., 2011), and they might also be useful for other drug-, gene- and cell-based therapeutic approaches. However, it is our consideration that the YG8sR mice could be of greatest use to investigate potential FRDA therapies that specifically target the human *FXN* gene sequence to increase frataxin expression, including DNA- or RNA-based oligotherapies (Sandi et al., 2013) and TALE-VP64 targeting of the *FXN* promoter (Chapdelaine et al., 2013). Owing to potential gender-specific effects of any particular therapy, we would propose to study both male and female YG8sR mice in such future therapeutic studies. In addition, the YG8sR mice will also be very useful to investigate any future strategies that might emerge to address the question of somatic GAA repeat instability and FRDA disease progression. In conclusion, we report a novel single-GAA-repeat-expansion-based FRDA mouse model that is available for investigators to work towards a therapy for this devastating disease.

## MATERIALS AND METHODS

### Transgenic mice, cell culture, DNA extraction and GAA repeat analysis

A founder YG8sR mouse containing ~120 GAA repeats was obtained by natural breeding of YG8R mice, and the new YG8sR line has been maintained on a predominantly C57BL6/J background. These mice are available from The Jackson Laboratory: YG8sR (#024113). Other mice are also available from The Jackson Laboratory: Y47R (#024097), YG8R (#012253) and YG22R (#012910). Mice were housed in conventional open cages with Litaspen Premium 8/20 bedding, paper wool nesting and standard fun tunnel environmental enrichment. The animal husbandry was carried out at 11 hour dark versus 13 hour light, 20–23°C and 45–60% humidity. The mice were nourished with a diet of SDS RM3 expanded food pellets and standard drinking water. All procedures were carried out in accordance with the UK Home Office ‘Animals (Scientific Procedures) Act 1986’ and with approval from the Brunel University Animals Welfare and Ethical Review Board. Mouse fibroblast cell lines were established from the kidneys of B6 mice and *FXN* YAC transgenic mouse models as previously described (Sandi et al., 2014). Genomic DNA was extracted from the FRDA and control mouse tissues and fibroblast cells by standard phenol/chloroform extraction and ethanol precipitation, and GAA PCR amplification was carried out using GAA-F (5′-GGGATTGGTTGCCAGTGCTTAAAAGTTAG-3′) and GAA-R (5′-GATCTAAGGACCATCATGGCCACACTTGCC-3′) primers (Campuzano et al., 1996), followed by sizing on agarose gels, as previously described (Al-Mahdawi et al., 2004). To determine YG8sR GAA repeat purity, *Mbo*II digestion of GAA PCR products and subsequent agarose gel electrophoresis was carried out as previously described (Holloway et al., 2011). To sequence the YG8sR GAA repeat, genomic DNA was extracted from fibroblast cells using the DNeasy Blood and Tissue kit (Qiagen), PCR amplification was performed on 50 ng of DNA using Q5 High Fidelity DNA polymerase (NEB) and primers 104F and 629R as previously described (Bidichandani et al., 1998), and Sanger sequencing was carried out with primer 629R.

### Estimation of transgene copy number

#### TaqMan copy number assays

The frataxin copy number was determined using TaqMan copy number assays (Applied Biosystems) according to the manufacturer’s instructions. In brief, 20 ng of genomic DNA was combined with 2× TaqMan universal master mix, TaqMan copy number assay for human *FXN* (Hs05092416_cn or Hs02407730_cn), and TaqMan copy number reference assay for mouse Tert in a 20 μl reaction volume. The assay was performed using the 7900-HT real-time polymerase chain reaction system and the following thermal cycling conditions: 50°C for 2 minutes, 95°C for 10 minutes, and 40 cycles of 95°C for 15 seconds and 60°C for 1 minute. Samples were assayed using triplicate wells for each gene of interest and copy numbers were estimated by relative quantitation (RQ) normalised to the known copy number of the reference sequence using the comparative Ct (ΔΔCt) method. The Ct data were subsequently compared to a calibrator sample known to have two copies of the target sequence, analysed by Applied Biosystems CopyCaller Software (v.2.0; Applied Biosystems) according to the product instruction.

#### Fluorescence *in situ* hybridisation assay

Cell cultures were harvested after exposure to colcemid for 4 hours and chromosome preparations were obtained according to standard cytogenetic methods. However, for interphase analysis, samples were produced without colcemid treatment. Cells were spread onto slides and were then denatured in 70% formamide in 2× SSC at 70°C for 5 minutes. The probes were prepared using purified DNA from RP11-265B8 and RP11-876N18 BAC clones, which were labelled by nick translation with biotin and digoxigenin, respectively, according to the manufacturer’s instructions (Roche). The labelled DNAs were ethanol precipitated together with Cot-1 human DNA (Roche) and resuspended in 10 μl of hybridisation buffer (Sigma). The probes were incubated at 65°C for 10 minutes, followed by preannealing at 37°C for 10 minutes. Hybridisation was carried out at 37°C overnight followed by washing with 2× SSC for 5 minutes. The RP11-265B8 probe was detected with Avidin D-Texas Red, biotinylated anti-Avidin D and Avidin D-Texas Red (Vector Laboratories). The RP11-876N18 was detected with mouse anti-digoxigenin antibody (Sigma-Aldrich) followed by rabbit anti-mouse FITC and anti-rabbit FITC (Sigma-Aldrich). The slides were mounted in VECTASHIELD (Vector Laboratories, Burlingame, CA, USA) containing DAPI counterstain.

### Behavioural testing

#### Body weight analysis and rotarod test

Weighing and rotarod tests were performed once a month from 4–12 months of age, using ten mice (five males and five females) from each group (B6, Y47R and YG8sR). Motor coordination ability was assessed using a Ugo-Basille 7650 accelerating rotarod treadmill. Four trials were performed with the speed of the rotation gradually increasing from 4 to 40 rpm and each trial lasted approximately 3 to 5 minutes, separated by a rest period of 200 seconds between each trial. The time taken for the mouse to fall from the apparatus was recorded and the maximum time on the rotarod was set at 400 seconds.

#### Beam-breaker locomotor activity test

Average velocity, ambulatory distance, vertical counts, vertical time, jump counts and jump time were measured over a 1-minute period and repeated five times for each mouse using a beam-breaker activity monitor (MEDOFA-510 activity chamber; Med Associates). Locomotor activity of the mice (*n*=10 including five males and five females used for each group) was assessed monthly over an 8-month period from 4 to 12 months of age. Data analysis and manipulation was performed using Microsoft Excel.

#### Beam-walk test

The beam-walk test was carried out using 90-cm long, 12-mm and 22-mm diameter, horizontal wooden beams. Coordination ability was assessed by measuring the time taken for the mouse to cross the beam. Mice received two trainings and were assessed four times on each beam with a rest period of 5 minutes between each trial.

#### Hang wire test

The hang wire test was performed to assess forelimb grip strength. The mice were hung from a horizontally positioned wire (2 mm in diameter and 30 cm long) with each end affixed to a vertical stand. Bedding material was placed underneath the wire to break the fall. The test commenced shortly after the mouse held onto the wire and the length of time before the fall was recorded. Four trials were performed with a rest period of 5 minutes between each trial.

#### Grip strength test

The grip strength meter (Salter Brecknell Model 12 Spring Balance) was also used to assess the forelimb grip strength. The mice, held by the base of the tail, were allowed to freely grasp a metal bar attached to a strain meter. The peak force with which mice pulled the bar horizontally was measured in four trials with a rest period of 5 minutes between each trial.

#### Footprint test

To obtain the footprints, mouse paws were dipped in nontoxic water-based food dye. The mice were allowed to walk along a 40-cm long, 9.5-cm wide, gangway (with 7-cm-high side walls) with white paper lining the floor. All mice had one training run and were then given three trials. Three steps from the middle portion of each run with a total number of nine steps for each mouse were measured for left hind and front stride length, right hind and front stride length, fore base width (the width between the right and left forelimbs) and hind base width (the width between the right and left hindlimbs).

### Glucose- and insulin-tolerance tests

To determine fasting blood glucose levels, 1 mg/g glucose solution (D-Glucose; Sigma-Aldrich) was injected intraperitoneally into the mice after a 16-hour fasting period. Blood glucose was measured from the tail vein immediately prior to glucose administration and after 20, 40 and 60 minutes with a glucometer (ACCU-CHEK Aviva blood glucose meter; Roche). For insulin-tolerance testing, the mice were fasted for 16 hours. Blood glucose was first measured from the tail vein, then the mice received an intraperitoneal injection of insulin (0.75 U/kg; Sigma-Aldrich) and blood glucose was measured again at time points of 20, 50 and 80 minutes after injection.

### Quantitative reverse transcriptase PCR

Total RNA was isolated from the mouse tissues by homogenisation with Trizol (Invitrogen) and cDNA was then prepared by using AMV reverse transcriptase (Invitrogen) with oligo(dT)_20_ primers following the manufacturer’s instructions. Levels of human transgenic *FXN* or endogenous *Fxn* mRNA expression were assessed by qPCR using an ABI Prism 7900HT Sequence Detection System and SYBR^®^ Green (Applied Biosystems) with the following primers that equally amplify human and mouse sequences: FRT-I forward 5′-TTGAAGACCTTGCAGACAAG-3′ and RRT-II reverse 5′-AGCCAGATTTGCTTGTTTGG-3′, 121-bp amplicon size. Mouse *Gapdh* RT-PCR primers used for normalisation were as follows: Gapdhm forward 5′-ACCCAGAAGACTGTGGATGG-3′ and Gapdhm reverse 5′-GGATGCAGGGATGATGTTCT-3′, 81-bp amplicon size. Assays were performed in triplicate in at least two independent experiments. *FAST-1* expression levels were determined as previously described (Sandi et al., 2014).

### Frataxin dipstick assay

Protein concentration was quantified by BCA assay and the levels of frataxin protein were measured by lateral flow immunoassay with the Frataxin Protein Quantity Dipstick Assay Kit (MitoSciences, Eugene, OR, USA) according to the manufacturer’s instructions (Willis et al., 2008). Signal intensity was measured with a Hamamatsu ICA-1000 Immunochromatographic Reader (MitoSciences).

### Histology

Histological preparations of mouse DRG were carried out by dissection of paraformaldehyde-fixed intact lumbar vertebrae, followed by decalcification treatment in Hillman and Lee’s EDTA daily for 5 days. Tissues were then embedded in paraffin wax, sectioned by standard methods, deparaffinized with IMS and Histoclear (National Diagnostics), and slides were stained with H&E.

### Aconitase assay

Aconitase activities were determined using the Aconitase Assay Kit (Cayman Chemical Company, 705502). Cell protein lysates (50 μl) were added to 200 μl of substrate mix [50 mM Tris/HCl pH 7.4, 0.4 mM NADP, 5 mM Na citrate, 0.6 mM MgCl_2_, 0.1% (v/v) Triton X-100 and 1 U isocitrate dehydrogenase] and the reactions were incubated at 37°C for 15 minutes, followed by spectrophotometric absorbance measurements at 340 nm at 37°C every minute for 15 minutes and subsequent determination of the reaction slope. Aconitase activities of mouse cells were then normalized to citrate synthase activities, which were determined using a citrate synthase assay kit (Sigma, CS0720).

### Statistical analyses

For statistical analysis, the unpaired two-tailed Student’s *t*-test or the two-way analysis of variance (ANOVA) test were used to assess the significance of the differences between group data with a significance value set at *P*<0.05.

## Supplementary Material

Supplementary Material
