# Supplementary material for: A novel GAA-repeat-expansion-based mouse model of Friedreich’s ataxia
Source: Dis Model Mech. 2015 Feb 13;8(3):225–35. doi: 10.1242/dmm.018952 (PMC4348561; doi:10.1242/dmm.018952)
Supplement: Supplementary Material [file supp_8_3_225__index.html]

A novel GAA-repeat-expansion-based mouse model of Friedreich’s ataxia — Supplementary Material 

# A novel GAA-repeat-expansion-based mouse model of Friedreich’s ataxia

## DMM018952 Supplementary Material

**Files in this Data Supplement:**

- **Supplementary Material**
