# Supplementary material for: A novel GAA-repeat-expansion-based mouse model of Friedreich’s ataxia
Source: Dis Model Mech. 2015 Feb 13;8(3):225–35. doi: 10.1242/dmm.018952 (PMC4348561; doi:10.1242/dmm.018952)
Supplement: Supplementary Material [file supp_8.3.225_DMM018952.pdf]

Supplementary data

Figure S1. Intergenerational GAA repeat instability. GAA PCR products from YG8 (five GAA bands shown here – numbers vary from two to five), YG8s (one GAA band), KIKO (one GAA band) and YG22 (two GAA bands) FRDA mice resolved on an agarose gel, together with 1kb+ and 100bp DNA size markers, showing intergenerational increases in YG8s GAA repeats from the original founder size of 120 GAA repeats to the current size of 200 GAA repeats.

Figure S2. MboII digestion and GAA repeat sequence analysis. (A) MboII digests and uncut controls of GAA PCR products from two YG8sR mice using GAA-F and GAA-R primers (Campuzano et al., 1996), together with a 1kb+ DNA size marker, showing complete digestion of the approximately 810bp PCR products, containing 120 GAA repeats, to leave 2 distinct bands consisting of the left and right non-GAA flanking sequences (C=cut with MboII, U=uncut, M=marker). (B) Representative electropherogram obtained by sequencing a GAA repeat PCR product from a YG8sR fibroblast cell line showing 133 pure GAA repeats.

Figure S3. Ambulatory distance. (A) YG8sR displayed significantly decreased ambulatory distance compared to B6 and Y47R controls when both male and female values were taken together (*n*=10 mice per genotype) or (B) males separately (*n*=5 mice per genotype). However, (C) YG8sR females had greater ambulatory distance; possibly due to their lower body weight. Values represent mean ± SEM.

Figure S4. Vertical time and counts. (A) Vertical time and (D) counts were significantly decreased in YG8sR compared to B6 and Y47R controls when both male and female values were analysed together (*n*=10 mice per genotype). Analysis of (B, E) males and (C, F) females separately (*n*=5 mice per genotype) revealed the same pattern. Values represent mean ± SEM.

Figure S5. Jump time and counts. (A) Jump time and (D) count were significantly decreased in YG8sR compared to B6 and Y47R controls when both male and female values were taken together (*n*=10 mice per genotype). Analysis of (B, E) males and

(C, F) females separately (n=5 mice per genotype) revealed the same pattern. Values represent mean ± SEM.

Figure S6. Footprint analysis. (A-C) Stride length (average of both left and right hindlimb, and left and right forelimb) analysis of mice. (A) Analysis of YG8sR mice revealed significantly reduced stride length compared to B6 and Y47R controls when both male and female values were taken together (n=10 mice per genotype). Analysis of (B) males and (C) females separately (n=5 mice per genotype). (D-F) Base width (average of forelimb and hindlimb base width) analysis of mice. (D) Analysis of YG8sR mice revealed significantly shorter base width compared to B6 and Y47R controls when both male and female values were taken together (n=10 mice per genotype). Analysis of (E) males and (F) females separately (n=5 mice per genotype). Values represent mean ± SEM. $^{***}P<0.001$. Statistical differences between YG8sR mutant and B6 control mice are indicated by the top bar while the bottom bar indicates the differences between YG8sR mutant and Y47R control mice.

Figure S7. Somatic GAA repeat instability. A representative 1.5% agarose gel shows GAA repeat PCR products obtained by using GAA-F and GAA-R primers (Campuzano et al., 1996) on DNA samples from different somatic tissues (Tail (Ta), Brain (B), Cerebellum (C), Liver (L), Heart (H), Kidney (K), Pancreas (P)) of YG8sR male (Lanes 3 to 9) and female (Lanes 10 to 16) mice. $1kb^+$ and 100bp DNA ladders were used as the molecular marker.

Table S1. Two-way ANOVA analysis of locomotor activity in YG8sR FRDA mice

Locomotor Activity	Gender	Versus B6	Versus Y47R
**Ambulatory distance**	**Male and Female**	P=0.004	P=0.1
	**Male**	P=1.28E-14	P=2.39E-09
	**Female**	P=0.004	P=0.004
**Vertical Time**	**Male and Female**	P=1.88E-12	P=5.62E-05
	**Male**	P=4.42E-07	P=0.01
	**Female**	P=1.07E-06	P=0.001
**Vertical Count**	**Male and Female**	P=1.05E-16	P=0.0004
	**Male**	P=2.26E-12	P=0.001
	**Female**	P=7.08E-06	P=0.09
**Jump Time**	**Male and Female**	P=4.67E-05	P=0.0008
	**Male**	P=7.84E-10	P=5.17E-05
	**Female**	P=0.9	P=0.3
**Jump Count**	**Male and Female**	P=2.37E-09	P=2.41E-06
	**Male**	P=8.34E-14	P=5.05E-07
	**Female**	P=0.4	P=0.1

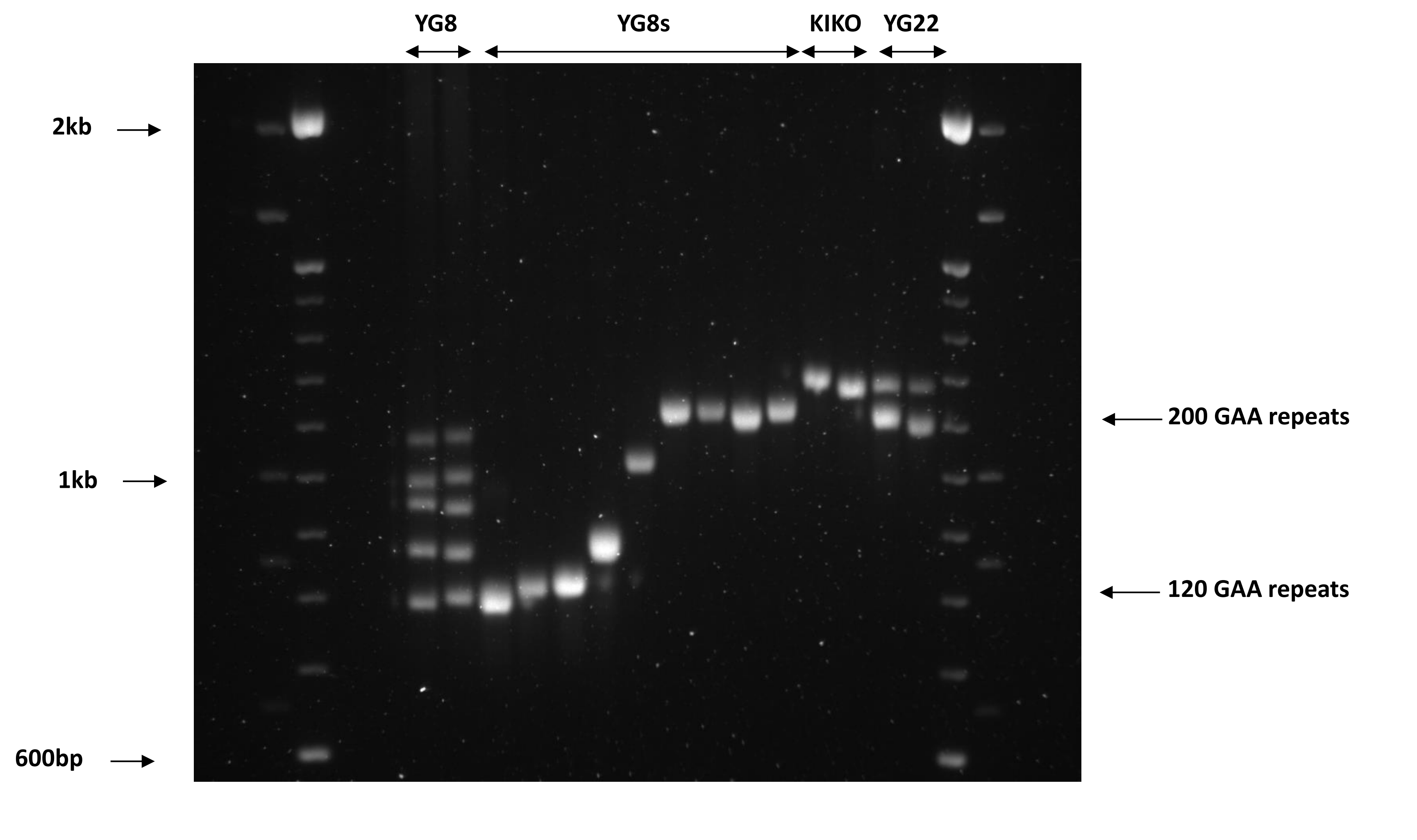
YG8
YG8s
KIKO
YG22
2kb
1kb
600bp
200 GAA repeats
120 GAA repeats

Figure S1.

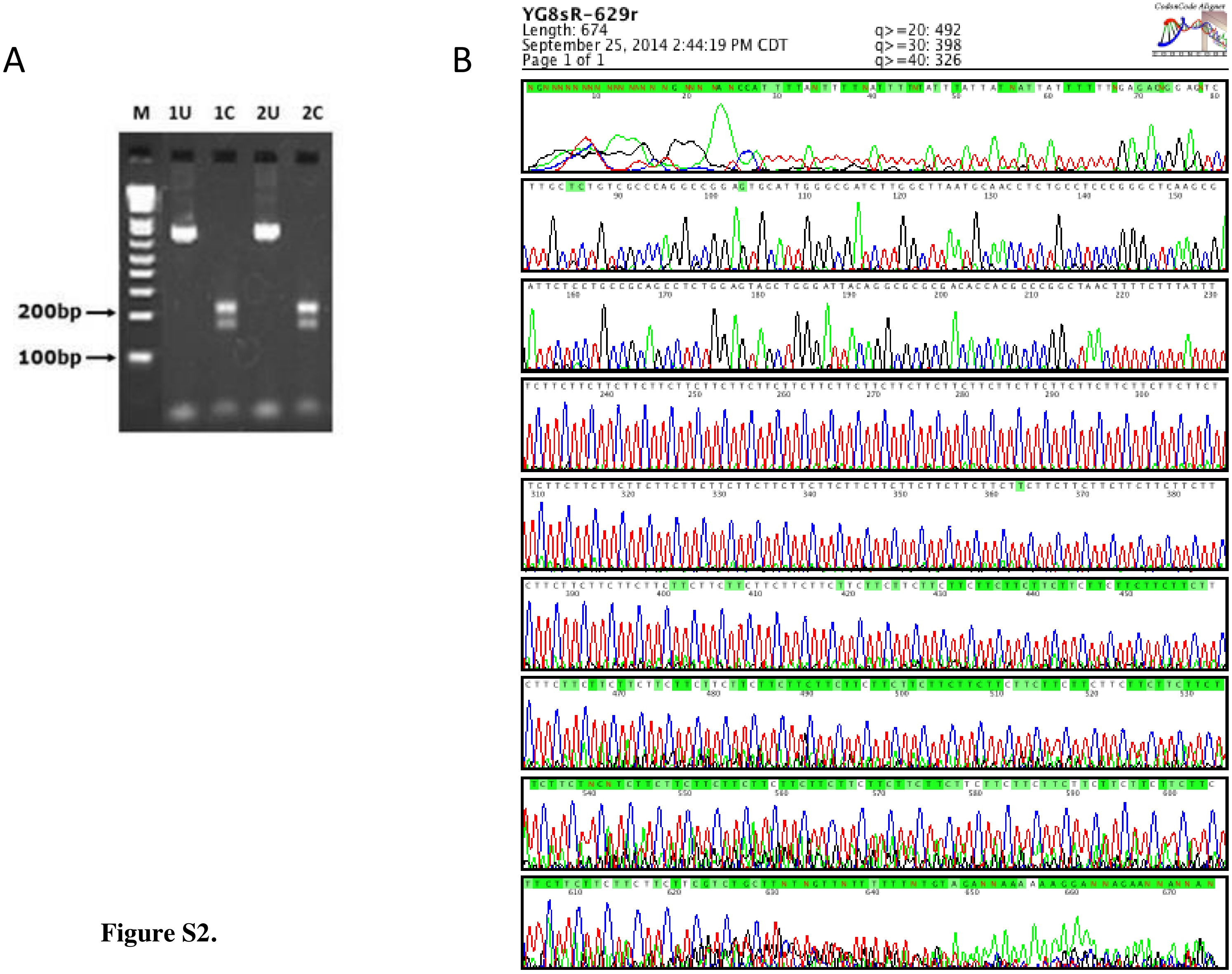
A
M
1U
1C
2U
2C
200bp
100bp
B
YG8sR-629r
Length: 674
September 25, 2014 2:44:19 PM CDT
Page 1 of 1
q>=20: 492
q>=30: 398
q>=40: 326

Figure S2.

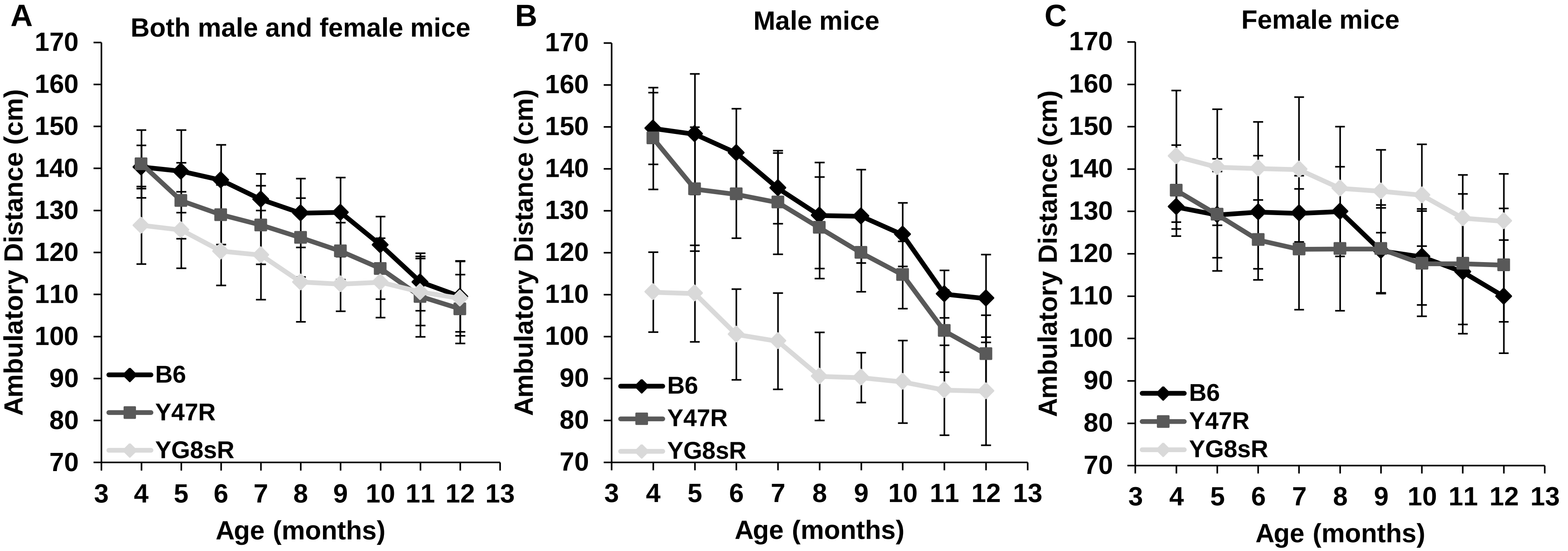
A
Both male and female mice
B
Male mice
C
Female mice
Ambulatory Distance (cm)
Age (months)
B6
Y47R
YG8sR
170
160
150
140
130
120
110
100
90
80
70
3
4
5
6
7
8
9
10
11
12
13

Figure S3.

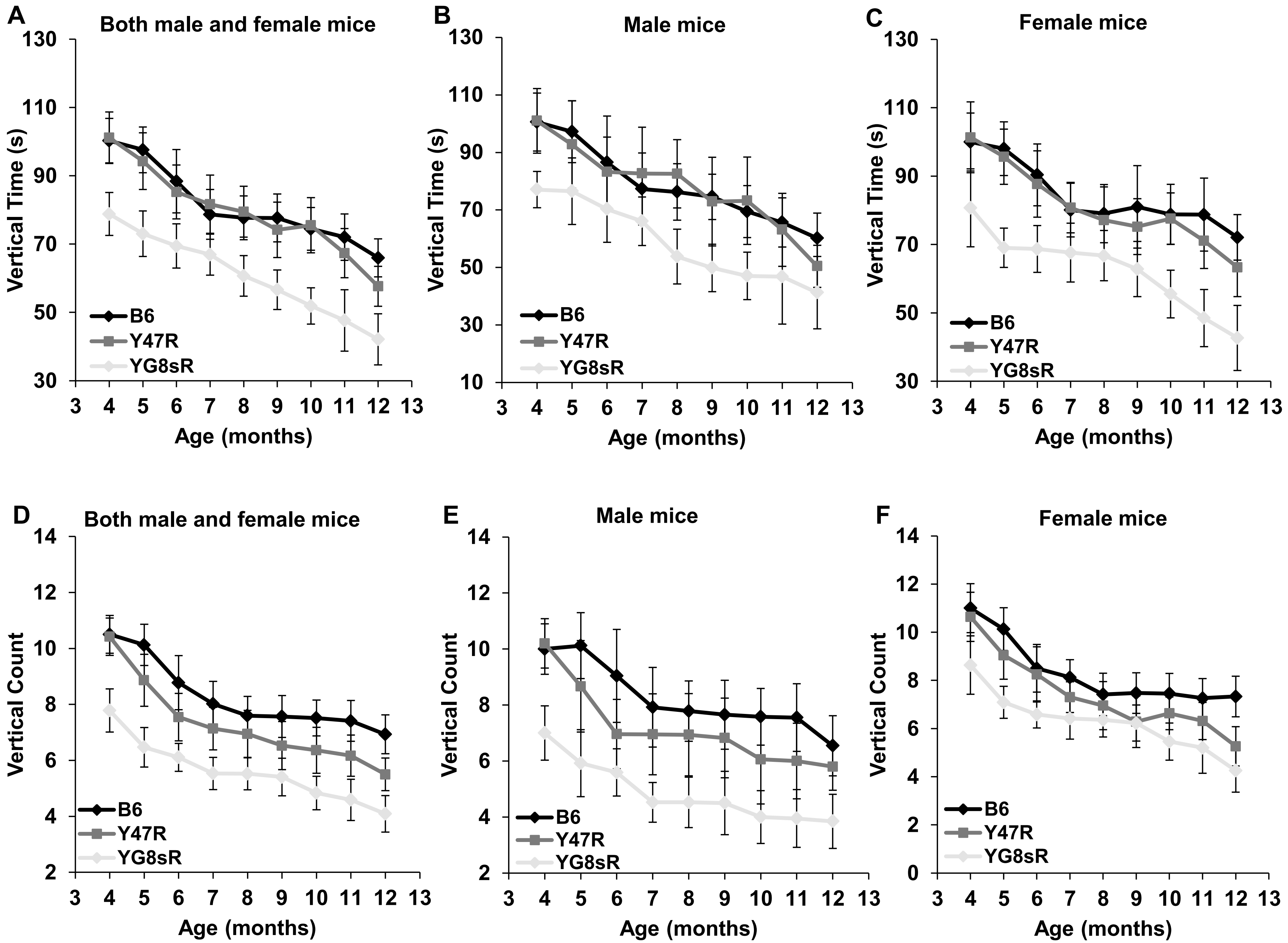
A
Both male and female mice
Vertical Time (s)
Age (months)
B6
Y47R
YG8sR
B
Male mice
Vertical Time (s)
Age (months)
B6
Y47R
YG8sR
C
Female mice
Vertical Time (s)
Age (months)
B6
Y47R
YG8sR
D
Both male and female mice
Vertical Count
Age (months)
B6
Y47R
YG8sR
E
Male mice
Vertical Count
Age (months)
B6
Y47R
YG8sR
F
Female mice
Vertical Count
Age (months)
B6
Y47R
YG8sR

Figure S4.

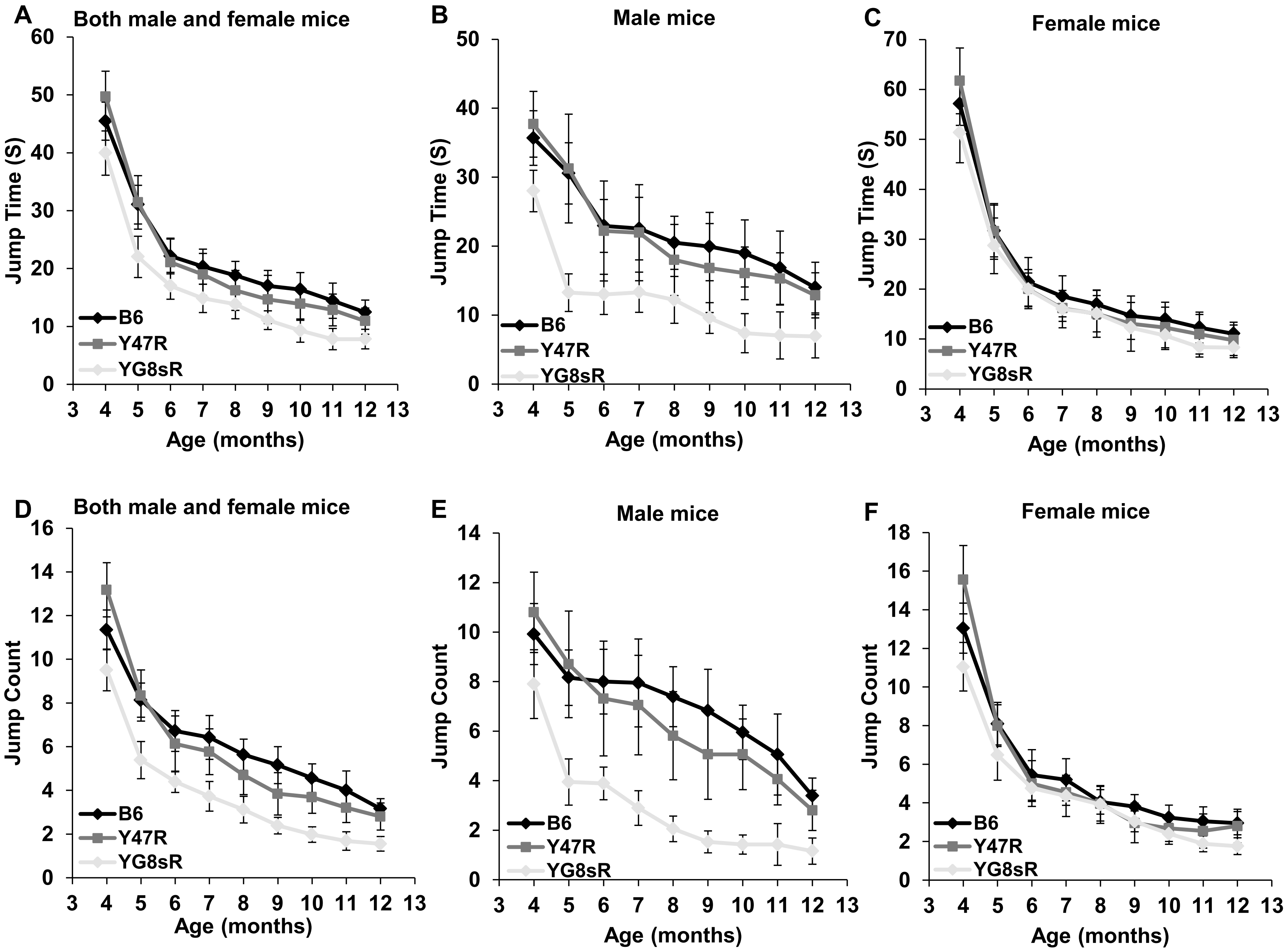
A
Both male and female mice
Jump Time (S)
Age (months)
B6
Y47R
YG8sR
B
Male mice
Jump Time (S)
Age (months)
B6
Y47R
YG8sR
C
Female mice
Jump Time (S)
Age (months)
B6
Y47R
YG8sR
D
Both male and female mice
Jump Count
Age (months)
B6
Y47R
YG8sR
E
Male mice
Jump Count
Age (months)
B6
Y47R
YG8sR
F
Female mice
Jump Count
Age (months)
B6
Y47R
YG8sR

Figure S5.

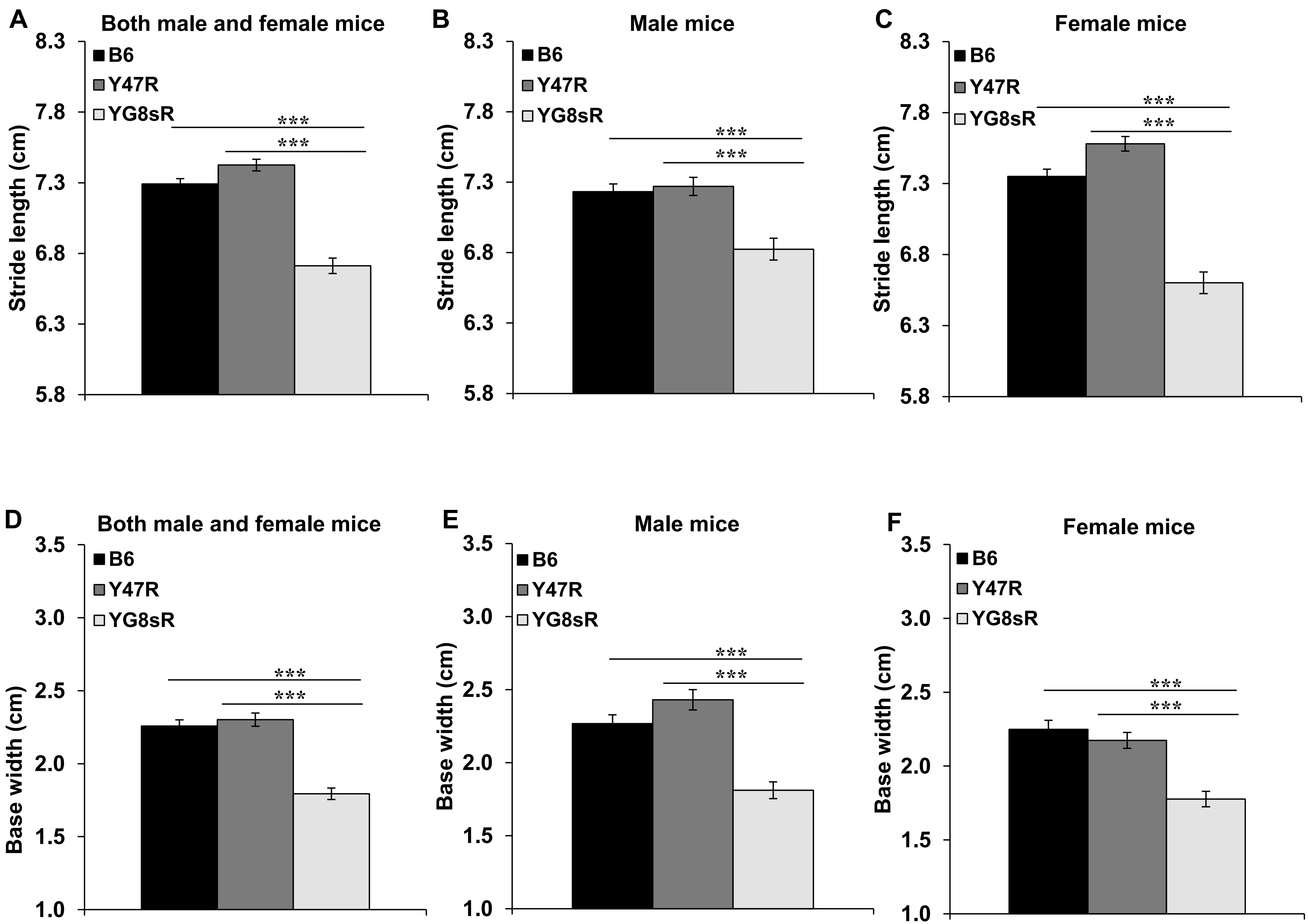
A
Both male and female mice
B
Male mice
C
Female mice
D
Both male and female mice
E
Male mice
F
Female mice
B6
Y47R
YG8sR
Stride length (cm)
Base width (cm)
***
8.3
7.8
7.3
6.8
6.3
5.8
3.5
3.0
2.5
2.0
1.5
1.0

Figure S6.

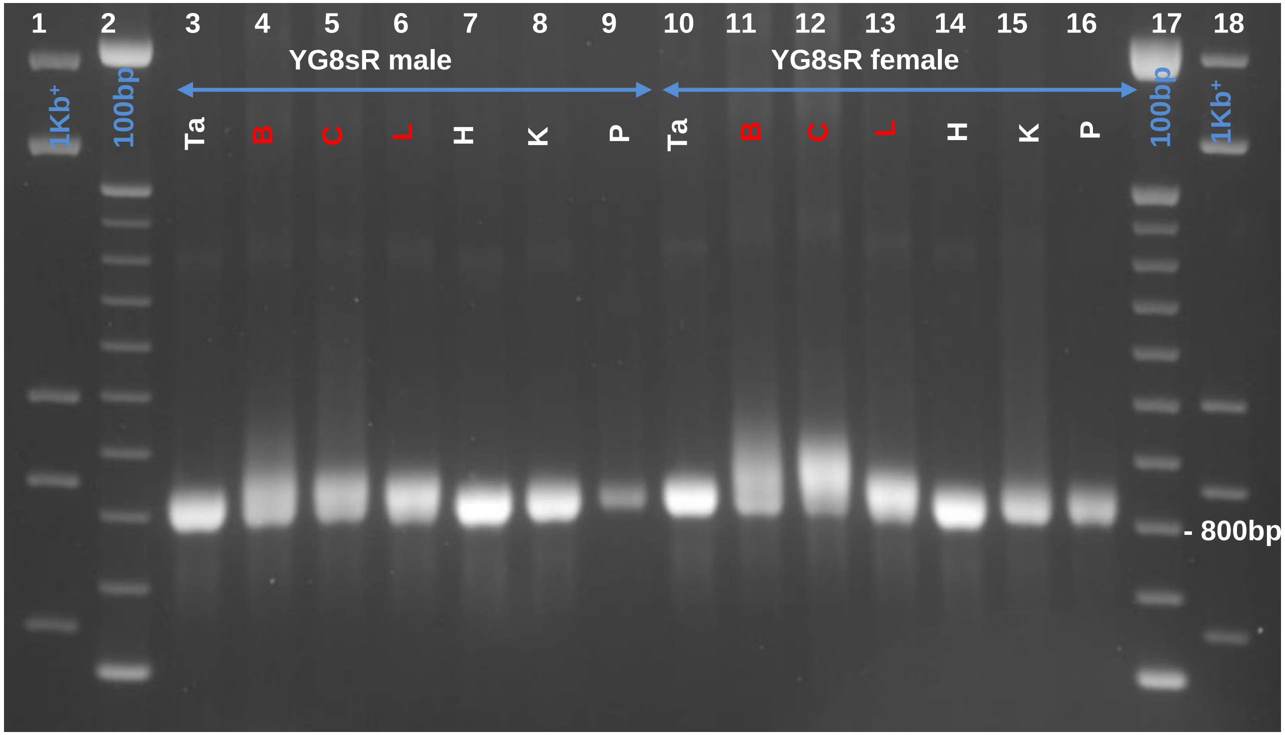
1
2
3
4
5
6
7
8
9
10
11
12
13
14
15
16
17
18
YG8sR male
YG8sR female
1Kb+
100bp
Ta
B
C
L
H
K
P
Ta
B
C
L
H
K
P
100bp
1Kb+
- 800bp

Figure S7.
